# Improved bounds for minimal feedback vertex sets in tournaments

**DOI:** 10.1002/jgt.22225

**Published:** 2017-12-08

**Authors:** M. Mnich, E. Teutrine

**Affiliations:** ^1^ Institut für Informatik Universität Bonn Bonn Germany; ^2^ Department of Quantitative Economics Maastricht University Maastricht The Netherlands

**Keywords:** combinatorial bounds, exponential‐time algorithms, feedback vertex sets, tournaments

## Abstract

We study feedback vertex sets (FVS) in tournaments, which are orientations of complete graphs. As our main result, we show that any tournament on *n* nodes has at most 1.5949^*n*^ minimal FVS. This significantly improves the previously best upper bound of 1.6667^*n*^ by Fomin et al. [STOC 2016] and 1.6740^*n*^ by Gaspers and Mnich [*J. Graph Theory* **72**(1):72–89, 2013]. Our new upper bound almost matches the best‐known lower bound of $21^{n/7}\approx 1.5448^{n}$, due to Gaspers and Mnich. Our proof is algorithmic, and shows that all minimal FVS of tournaments can be enumerated in time $O(1.5949^{n})$.

## 1 INTRODUCTION

The minimum feedback vertex set (fvs) problem in directed graphs is a fundamental problem in combinatorial optimization: given a directed graph *G*, find a smallest set of vertices in *G* whose removal yields an acyclic digraph. This problem belongs to Karp's original list of 21 NP‐hard problems 8.

The minimum fvs problem remains NP‐hard even in tournaments 14, which are orientations of complete undirected graphs. In other words, a tournament *T* is a digraph with exactly one arc between any two of its vertices. Various approaches have been suggested to solve the minimum fvs problem on tournaments, including approximation algorithms 3, 11, fixed‐parameter algorithms 4, 9 as well as exact exponential‐time algorithms 4, 5, 6. In particular, one approach that was used to find a minimum FVS is to list all inclusion‐minimal FVS of a given tournament using a polynomial‐delay enumeration algorithm 6, 13. The running time of this approach is within a polynomial factor of the number M(T) of minimal FVS in *T*. Therefore, using this approach, the complexity of the minimum fvs problem in tournaments is within a polynomial factor of the maximum of M(T) over all *n*‐vertex tournaments, which we denote by M(n).

The first one to provide nontrivial bounds on M(n) was Moon 12, who in 1971 established that $1.4757^{n}\leq M\left(n\right)\leq 1.7170^{n}$. This was improved by Gaspers and Mnich 6 in 2010 to $1.5448^{n}\leq M\left(n\right)\leq 1.6740^{n}$. Very recently, an improvement on the upper bound was made by Fomin et al. 5, who show that $M\left(n\right)\leq 1.6667^{n}$. The problem of exactly determining M(n) was explicitly posed by Woeginger 16.

### 1.1 Our contributions

In this article, we make significant progress on establishing better bounds for M(n). Our main combinatorial result is as follows:

Theorem 1 Any tournament of order *n* has at most $M\left(n\right)\leq 1.5949^{n}$ minimal FVS.

We also consider regular tournaments (in which all vertices have the same out‐degree), because the best‐known lower bound on M(n) is attained by regular tournaments. For regular tournaments, we show an upper bound on M(n) that matches the lower bound:

Theorem 2 Any regular tournament of order *n* has at most $21^{n/7}$ minimal FVS, and this is sharp: some regular tournament of order *n* has exactly $21^{n/7}$ minimal FVS.

The following Table 1 provides an overview on lower and upper bounds on M(n):

**Table 1:** State of the art for lower and upper bounds on the number of minimal FVS in tournaments

M(n)	Lower Bound	Upper Bound
Moon (1971)	1.4757^*n*^	1.7170^*n*^
Gaspers and Mnich (J. Graph Theory, 2013)	$21^{n/7}\approx 1.5448^{n}$	1.6740^*n*^
Fomin et al. (STOC 2016)		1.6667^*n*^
*This article*		1.5949^*n*^
*This article, regular tournaments*:		$21^{n/7}\approx 1.5448^{n}$

Our proof of Theorem 1 is inspired by the one of Gaspers and Mnich 6 for their weaker upper bound. Their proof works by induction on the number *n* of nodes in the input tournament *T*. Starting with *T*, they consider a vertex *v* with maximum out‐degree Δ, and depending on the value of Δ and neighbors of *v*, they construct subtournaments by deleting distinct vertices, such that each maximal transitive vertex set of *T* is contained in at least one subtournament. Applying the induction hypothesis to the subtournaments then implies their upper bound.

Here, we use a refined technique, that yields upper bounds on the number of inclusion‐maximal vertex sets with certain properties. Namely, in addition to deleting vertices to generate subtournaments, we also keep fixed vertex sets. Within these subtournaments we only consider maximal transitive vertex sets that contain all the fixed vertices. We introduce a new function M(n,k) for the maximum number of maximal transitive vertex sets in a tournament of order *n* containing a fixed set of *k* vertices, and we will show that $M\left(n,k\right)\leq 1.5949^{n-k}$ for all 0≤k≤n. A similar approach has been used by Gupta et al. 7 to bound the number of maximal *r*‐regular induced subgraphs in undirected graphs.

Our combinatorial result has algorithmic consequences. First, our proof of Theorem 1 is algorithmic, and shows that all minimal FVS of any tournament of order *n* can be listed in time $O(1.5949^{n})$. Second, using an algorithm by Gaspers and Mnich 6 to list all minimal FVS of a tournament with polynomial delay and in polynomial space, we directly obtain the following:

Corollary 1 Given any tournament *T* of order *n*, all its minimal FVS can be listed in time $M\left(T\right)\;\cdot n^{O(1)}=O\left(1.5949^{n}\right)$ with polynomial delay and in polynomial space.

Enumerating the minimal FVS in tournaments has several interesting applications. For example, Banks 1 introduced the notion “Banks winner” in a social choice context, which is a vertex *v* with in‐degree 0 in a subtournament induced by a maximal transitive vertex set. Brandt et al. 2 consider the problem of determining the “Banks set,” which is the set of all Banks winners. As Woeginger 15 showed that deciding whether a vertex is a Banks winner is NP‐complete, a feasible approach to determine the Banks set is to enumerate all minimal FVS. For this purpose, Brandt et al. 2 implemented the algorithm of Gaspers and Mnich. Thus, our new algorithm in this article yields an improved worst‐case bound on the time to compute the Banks set of tournaments.

## 2 PRELIMINARIES

A *tournament* T=(V,A) is a directed graph with exactly one edge between each pair of vertices. We denote the set of all tournaments with *n* vertices by $T_{n}$. A *feedback vertex set* (FVS) of *T* is a set F⊆V(T) such that T−F is free of (directed) cycles, where T−F is the induced subgraph of *T* after removing all vertices in *F*. An FVS is *minimal* if none of its proper subsets is an FVS.

Denote by M(T) the number of minimal FVS in a tournament *T*, and define

$$\begin{array}{c} M\left(n\right)=\max_{T\in T_{n}}M\left(T\right) \end{array}$$

to be the maximum number of minimal FVS in tournaments of order *n*.

Let T=(V,A) be a tournament. For a set $V^{\prime }\subseteq V$, let $T[V^{\prime }]$ be the subtournament of *T* induced by $V^{\prime }$. For each v∈V, let $N^{-}\left(v\right)=\left\{u\in V\;|\;\left(u,v\right)\in A\right\}$ and let $N^{+}\left(v\right)=\left\{u\in V\;|\;\left(v,u\right)\in A\right\}$. We write v→u if $u\in N^{+}\left(v\right)$ and call *v* a *predecessor* of *u* and *u* a *successor* of *v*. For each v∈V, its *in‐degree* is $d^{-}\left(v\right)=\left|N^{-}\left(v\right)\right|$ and its *out‐degree* is $d^{+}\left(v\right)=\left|N^{+}\left(v\right)\right|$; call *T* *regular* if all its vertices have the same out‐degree. Let $\Delta^{+}\left(T\right)$ denote the maximum out‐degree over all vertices of *T*. Further, *T* is *strong* if there is a directed path from *v* to *u* for each pair of vertices v,u∈V; let $T_{n}^{★}$ denote the set of strong tournaments of order *n*. Note that any tournament can uniquely be decomposed into strong subtournaments $S_{1},\cdots ,S_{r}$ such that v→u for all $v\in V(S_{i})$, $u\in V(S_{j})$ for all i<j.

Observation 1 For any tournament *T*, we obtain $M\left(T\right)=M\left(S_{1}\right)\cdot \cdots \cdot M\left(S_{r}\right)$.

Therefore, we can bound M(n) from above by $\beta^{n}$ for some β by considering strong tournaments of every order *n*.

Our proofs will use the following well‐known observation about cycles in tournaments:

Lemma 1 In a tournament, any vertex contained in a cycle is contained in a directed triangle.

Let $v_{1},\cdots ,v_{\ell }$ be a shortest cycle containing *v*_1_ with ℓ>3, $v_{i}\to v_{i+1}$ for all i∈{1,⋯,ℓ−1} and $v_{\ell }\to v_{1}$. Depending on the orientation of the arc between *v*_1_ and *v*_3_, either $v_{1},v_{2},v_{3}$ form a triangle or $v_{1},v_{3},v_{4},\cdots ,v_{\ell }$ is a shorter cycle containing *v*_1_. ▪

Henceforth, throughout the article by “triangle” we always mean “directed triangle.”

We call a vertex set *transitive* if its induced subtournament is acyclic. Thus, a vertex set is a maximal transitive vertex set if and only if its complement is a minimal FVS. Instead of counting minimal FVS, we count maximal transitive vertex sets. The next property of maximal transitive vertex sets was already used by Moon 12 and Gaspers and Mnich 6:

Lemma 2 For any tournament *T*, $M\left(T\right)\leq \sum_{v\in V(T)}M\left(d^{+}\left(v\right)\right)$.

Any maximal transitive vertex set *W* of *T* has a vertex *v* with in‐degree 0 in T[W]. Hence, *W* is also a maximal transitive vertex set in $T[N^{+}\left(v\right)\cup \left\{v\right\}]$; this yields the bound. ▪

Lemma 2 allows us to effectively bound M(T) in terms of a recurrence relation, in particular in combination with the next lemma that extends Lemma 3 by Gaspers and Mnich 6:

Lemma 3 Let n∈N and let $T\in T_{n}^{★}$. Then either *T* is regular, or for any d∈N at most 2*d* vertices in *T* have out‐degree at least n−d−1.

Let $\tilde{V}$ be the set of vertices in *T* with out‐degree at least n−d−1. Then any vertex in $\tilde{V}$ has in‐degree at most *d*. Hence,

(1) $$\sum_{v\in \tilde{V}}|N^{-}\left(v\right)|\leq |\tilde{V}|\cdot d.$$

We may suppose that $\tilde{V}\neq \emptyset$, for otherwise the statement of the lemma holds. We distinguish two cases.

Consider first the case that $\tilde{V}\neq V\left(T\right)$. Then, since *T* is strong and $\tilde{V}\neq \emptyset$, there is some arc from $V\left(T\right)\setminus \tilde{V}$ to $\tilde{V}$. There are |V∼|2 arcs between vertices in $\tilde{V}$. Therefore, ∑v∈V∼|N−(v)|≥|V∼|2+1. Combining this inequality with (1) and solving for d∈N yields $|\tilde{V}|\leq 2d$.

Second, consider the case that $\tilde{V}=V\left(T\right)$. We may suppose that *T* is not regular, for otherwise the statement of the lemma holds. Note that not every vertex of $\tilde{V}=V\left(T\right)$ can have in‐degree exactly *d*, since *T* is not regular. Hence, some vertex in $\tilde{V}$ has in‐degree at most d−1. Consequently,

$$\sum_{v\in \tilde{V}}|N^{-}\left(v\right)|\leq (|\tilde{V}\left|-1\right)\cdot d+\left(d-1\right).$$

There are |V∼|2 arcs between vertices in $\tilde{V}$. Thus, ∑v∈V∼|N−(v)|≥|V∼|2. Combining these two inequalities and solving for d∈N yields $|\tilde{V}|\leq 2d$. ▪

We remark that a regular tournament may have more than 2*d* vertices of out‐degree at least n−d−1, as witnessed for instance by the triangle and d=1.

## 3 IMPROVED UPPER BOUND ON THE MAXIMUM NUMBER OF MINIMAL FVS

In this section, we show that the maximum number M(n) of minimal FVS in any tournament of order *n* is bounded from above by 1.5949^*n*^. For this purpose, for a tournament *T* and $V^{\prime }\subseteq V\left(T\right)$ let $M(T,V^{\prime })$ be the number of maximal transitive vertex sets in *T* that contain all vertices in $V^{\prime }$. Also, let

$$\begin{array}{c} M\left(n,k\right)=\max_{T\in T_{n},V^{\prime }\subseteq V\left(T\right),\left|V^{\prime }\right|=k}M\left(T,V^{\prime }\right). \end{array}$$

Note that M(n)=M(n,0).

**Example**. To clarify the definition, we compute *M*(3, 1). Precisely, we show that M(3,1)=2. There are two nonisomorphic tournaments for n=3:


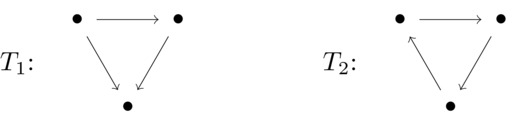


The tournament *T*_1_ is acyclic and thus has only a single maximal transitive vertex set, $V(T_{1})$. Thus, $M(T_{1},\left\{v\right\})=1$ for all $v\in V(T_{1})$. The tournament *T*_2_ has three maximal transitive vertex sets, each consisting of exactly two vertices. Thus, each vertex of *T*_2_ is contained in exactly two maximal transitive vertex sets. This yields $M(T_{2},\left\{v\right\})=2$ for all $v\in V(T_{2})$. Summarizing, we get M(3,1)=2.

Henceforth, fix β=1.5949. We will show that $M\left(n,k\right)\leq \beta^{n-k}$ for all n∈N and k∈{0,⋯,n}. To this end, ideally we would like to prove the following statements:

(I) *It holds* $M\left(n,k\right)\leq \beta^{n-k}$ *for all* n≥k>0.
(II) *It holds* $M\left(n,0\right)\leq \beta^{n}$.

Unfortunately, we are unable to do prove these directly. The reason is that our proof of Statement (I) for a fixed pair (n,k) with n≥k>0 *depends* on the validity of Statement (II) for values $\tilde{n}<n$. Vice versa, our proof of the validity of Statement (II) for fixed n∈N depends on the validity of Statement (I).

We will therefore establish the following two lemmas:

Lemma 4 Let n∈N. If $M\left(\tilde{n}\right)\leq \beta^{\tilde{n}}$ and $M\left(\tilde{n},\tilde{k}\right)\leq \beta^{\tilde{n}-\tilde{k}}$ holds for all $0<\tilde{k}\leq \tilde{n}<n$, then $M\left(n,k\right)\leq \beta^{n-k}$ for 0<k≤n.

The proof of Lemma 4 is given in Section 4.

Lemma 5 Let n∈N. If $M\left(\tilde{n}\right)\leq \beta^{\tilde{n}}$, $M\left(\tilde{n},\tilde{k}\right)\leq \beta^{\tilde{n}-\tilde{k}}$ and $M\left(n,\tilde{k}\right)\leq \beta^{n-\tilde{k}}$ for all $0<\tilde{k}\leq \tilde{n}<n$, then $M\left(n\right)\leq \beta^{n}$.

The proof of Lemma 5 consists of a lengthy case analysis; we thus defer it to Section 5.

We are ready to prove Theorem 1.

Proof of Lemma 1 We show that for all n∈N, it holds $M\left(n\right)\leq 1.5949^{n}$. Clearly, M(1)≤1≤1.5949 and $M\left(1,k\right)\leq 1\leq 1.5949^{1-k}$ for all k∈{0,1}. This yields our induction hypothesis. Lemma 4 and Lemma 5 yield our inductive step and prove the desired bound on M(n) for all n∈N. ▪

## 4 PROOF OF LEMMA 4

In this section, we prove Lemma 4. For sake of contradiction, suppose that the statement of the lemma does not hold. Let $\left(T,V^{\prime }\right)$ be a minimum counterexample, that is, *T* is a tournament and $V^{\prime }\subseteq V\left(T\right)$ such that $\left|V\left(T\right)|-\right|V^{\prime }|$ is minimum and $M\left(T,V^{\prime }\right)>\beta^{\left|V\left(T\right)|-\right|V^{\prime }|}$. Throughout this section, write n=|V(T)| and $k=|V^{\prime }|>0$.

We will distinguish several cases and show that $M\left(T,V^{\prime }\right)\leq \beta^{n-k}$ for each of them; this yields the desired contradiction (and hence the truth of the statement of the lemma). In each case, we will use the minimality of $\left(T,V^{\prime }\right)$ to bound $M(T,V^{\prime })$ from above.

**Case 1**: Three vertices in $V^{\prime }$ form a triangle.
  Then, as no transitive vertex set contains all of these three vertices, $M\left(T,V^{\prime }\right)=0\leq \beta^{n-k}$.
**Case 2**: Two vertices in $V^{\prime }$ form a triangle with some vertex $v\in V\left(T\right)\setminus V^{\prime }$.
  Any transitive vertex set that contains all vertices in $V^{\prime }$ does not contain *v*. Hence,
  $$\begin{array}{c} M\left(T,V^{\prime }\right)=M\left(T-\left\{v\right\},V^{\prime }\right)\leq M\left(n-1,k\right)\leq \beta^{n-k-1}\leq \beta^{n-k}. \end{array}$$
**Case 3**: There is a vertex $v\in V^{\prime }$ that is not contained in any cycle of *T*.
  Then, a set $W⊇V^{\prime }$ is a maximal transitive vertex set of *T* if and only if $W\setminus \left\{v\right\}⊇V^{\prime }\setminus \left\{v\right\}$ is a maximal transitive vertex set of T−v. This yields

$$\begin{array}{c} M\left(T,V^{\prime }\right)=M\left(T-\left\{v\right\},V^{\prime }\setminus \left\{v\right\}\right)\leq M\left(n-1,k-1\right)\leq \beta^{n-k}. \end{array}$$

Remark 1 We remark that it is this case where we rely on the validity of Lemma 5, namely that $M\left(\tilde{n}\right)<\beta^{\tilde{n}}$ for $\tilde{n}<n$. The reason is that possibly $V^{\prime }\setminus \left\{v\right\}=\emptyset$, in which case k−1=0 and we need that $M\left(n-1,0\right)\leq \beta^{n-1}$.

Henceforth, consider pairs $\left(T,V^{\prime }\right)$ to which Cases 1–3 do not apply.

Observation 2 If Cases 1–3 do not apply to $\left(T,V^{\prime }\right)$, then (i) any vertex of $V^{\prime }$ is contained in at least one triangle (by Lemma 1), and (ii) any triangle contains at most one vertex of $V^{\prime }$.

Remark 2 We remark that with Case 1–3 we can already show a bound of $M\left(T,V^{\prime }\right)\leq \beta_{0}^{n-k}$ for $\beta_{0}=1.6181$ (under the conditions imposed by the lemma). By  Observation 2, there is a vertex $v\in V^{\prime }$ that forms a triangle with two vertices $w_{1},w_{2}\notin V^{\prime }$. Any maximal transitive vertex set $W⊇V^{\prime }$ (and thus containing *v*) cannot contain both *w*_1_ and *w*_2_. Therefore, $w_{1}\in W$ implies $w_{2}\notin W$ and we get

$$\begin{array}{ccc} M(T,V^{\prime }) & \leq & M\left(T-\left\{w_{1}\right\},V^{\prime }\right)+M\left(T-\left\{w_{2}\right\},V^{\prime }\cup \left\{w_{1}\right\}\right)\\ & \leq & M\left(n-1,k\right)+M\left(n-1,k+1\right)\leq \beta_{0}^{n-k-1}+\beta_{0}^{n-k-2}, \end{array}$$

which is bounded by $\beta_{0}^{n}$ for $\beta_{0}=1.6181$.

The subsequent cases allow us to improve $\beta_{0}=1.6181$ to β=1.5949.

**Case 4**: There is a vertex $w\notin V^{\prime }$ that is contained in two distinct triangles, both of which contain a vertex from $V^{\prime }$ (possibly shared by both triangles). Then we are in one of two cases, where vertices in $V^{\prime }$ are circled:

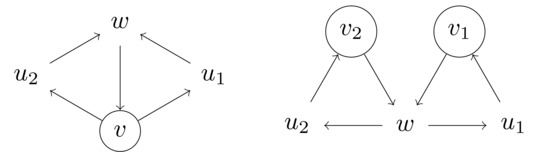


Let $\left(w,u_{1},v_{1}\right),\left(w,u_{2},v_{2}\right)$ be distinct triangles containing *w*, such that $v_{1},v_{2}\in V^{\prime }$ where possibly $v_{1}=v_{2}$. Let *W* be a maximal transitive vertex set of *T* containing $V^{\prime }$. Then either w∉W or w∈W. Clearly, if w∈W then $u_{1},u_{2}\notin W$. We therefore have

$$\begin{array}{ccc} M(T,V^{\prime }) & \leq & M\left(T-\left\{w\right\},V^{\prime }\right)+M\left(T-\left\{u_{1},u_{2}\right\},V^{\prime }\cup \left\{w\right\}\right)\\ & \leq & M\left(n-1,k\right)+M\left(n-2,k+1\right)\leq \beta^{n-k-1}+\beta^{n-k-3}. \end{array}$$

The last expression on the right‐hand side is at most $\beta^{n-k}$, since β≥1.4656.

**Case 5**: There are vertices $v\in V^{\prime }$ and $w_{1},w_{2}\in V\left(T\right)\setminus V^{\prime }$ that form a triangle, such that *w*_1_ also belongs to triangles $\left(w_{1},u_{1},u_{2}\right),\left(w_{1},u_{2},u_{3}\right)$ for some $u_{1},u_{2},u_{3}\in V\left(T\right)\setminus \left\{v,w_{2}\right\}$.

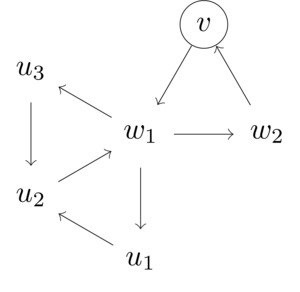


Then we can assume that $u_{1},u_{2},u_{3}\in V\left(T\right)\setminus V^{\prime }$, as otherwise Case 2 or Case 4 would apply. Any transitive vertex set $W⊇V^{\prime }$ either contains *w*_1_ or not. If $w_{1}\in W$ then $w_{2}\notin W$. Moreover, $w_{1}\in W$ implies that either $u_{2}\notin W$, or $u_{2}\in W$ but $u_{1},u_{3}\notin W$. Thus,

$$\begin{array}{ccc} M(T,V^{\prime }) & \leq & M\left(T-\left\{w_{1}\right\},V^{\prime }\right)+M\left(T-\left\{w_{2}\right\},V^{\prime }\cup \left\{w_{1}\right\}\right)\\ & \leq & M\left(T-\left\{w_{1}\right\},V^{\prime }\right)+M\left(T-\left\{w_{2},u_{2}\right\},V^{\prime }\cup \left\{w_{1}\right\}\right)\\ & & +M(T-\left\{w_{2},u_{1},u_{3}\right\},V^{\prime }\cup \left\{w_{1},u_{2}\right\})\\ & \leq & M(n-1,k)+M(n-2,k+1)+M(n-3,k+2)\\ & \leq & \beta^{n-k-1}+\beta^{n-k-3}+\beta^{n-k-5}. \end{array}$$

The last expression on the right‐hand side is at most $\beta^{n-k}$, since β≥1.5702.

Henceforth, we assume that Cases 1–5 do not apply to $\left(T,V^{\prime }\right)$. Then some vertex $v_{0}\in V^{\prime }$ forms a triangle with some $w_{1},w_{2}\in V\left(T\right)\setminus V^{\prime }$, as Cases 1–3 do not apply. For i=1,2, let $\Delta_{i}$ be the set of triangles $t_{i}=\left(u_{i},v_{i},w_{i}\right)$ that are disjoint from $w_{3-i}$ and for which $T[\left\{u_{i},v_{i},v^{\prime }\right\}]$ is acyclic for all $v^{\prime }\in V^{\prime }$. Consequently, all triangles in $\Delta_{1}\cup \Delta_{2}$ are disjoint from $V^{\prime }$, as Case 4 does not apply. Further, all triangles in $\Delta_{i}$ are pairwise edge‐disjoint (as Case 5 does not apply), and therefore intersect only in $w_{i}$.

To prove an upper bound on $M(T,V^{\prime })$, we again distinguish the maximal transitive vertex sets that contain *w*_1_ or *w*_2_, from those that do not contain either of them. Let *W* be a maximal transitive vertex set of *T* containing $V^{\prime }$.

First consider that $w_{1},w_{2}\notin W$. Then, $T[W\cup \left\{w_{i}\right\}]$ contains a cycle for i=1,2, by maximality of *W*. Thus, by Lemma 1, there is a triangle $t=(w_{i},z_{1},z_{2})$ for some $z_{1},z_{2}\in W$. We have that $t\in \Delta_{i}$, since $z_{1},z_{2}$ do not form a triangle with any $v^{\prime }\in V^{\prime }$ as $z_{1},z_{2}\in W$. Thus, those *W* with $w_{1},w_{2}\notin W$ can be partitioned into $|\Delta_{i}|$ classes, where the *r*‐th class contains the sets *W* that contain the two vertices of the *r*‐th triangle in $\Delta_{i}$.

To use this argument effectively, we need some further observations about the relation among triangles in $\Delta_{1}\cup \Delta_{2}$. Consider two triangles $t_{i}^{r}=\left(u_{i}^{r},v_{i}^{r},w_{i}\right),t_{i}^{s}=\left(u_{i}^{s},v_{i}^{s},w_{i}\right)\in \Delta_{i}$:


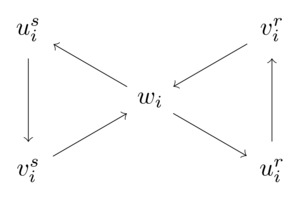


Since all triangles that contain $w_{i}$ are pairwise edge‐disjoint (as Case 5 does not apply), the edge between $u_{i}^{r}$ and $v_{i}^{s}$ has to be directed from $v_{i}^{s}$ to $u_{i}^{r}$; else, $w_{i},u_{i}^{r},v_{i}^{s}$ would form a triangle that is not edge‐disjoint from the triangle $w_{i},u_{i}^{r},v_{i}^{r}$. Likewise, the edge between $u_{i}^{s}$ and $v_{i}^{r}$ has to be directed from $v_{i}^{r}$ to $u_{i}^{s}$. Ignoring symmetries obtained by swapping the roles of $t_{i}^{r}$ and $t_{i}^{s}$, there are only two possibilities how the two remaining edges (between $u_{i}^{r},u_{i}^{s}$ and $v_{i}^{r},v_{i}^{s}$) can be oriented:


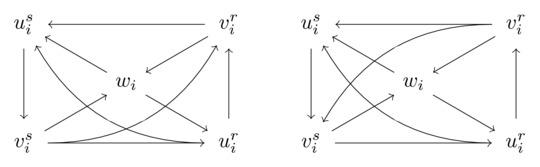


We refer to the situation in the left figure as **Case A**, and to the situation in the right figure as **Case B**. Note that in Case A, $\left(u_{i}^{r},u_{i}^{s},v_{i}^{s}\right)$ and $\left(v_{i}^{r},u_{i}^{s},v_{i}^{s}\right)$ form triangles; while in Case B, triangles are formed by $\left(u_{i}^{r},u_{i}^{s},v_{i}^{s}\right)$ and $\left(u_{i}^{r},v_{i}^{r},v_{i}^{s}\right)$.

Observation 3 In Case A, $u_{i}^{s},v_{i}^{s}\in W$ implies that $u_{i}^{r},v_{i}^{r}\notin W$. In Case B, $u_{i}^{r},v_{i}^{r}\in W$ implies that $v_{i}^{s}\notin W$; and $u_{i}^{s},v_{i}^{s}\in W$ implies that $u_{i}^{r}\notin W$.

Thus, for each $t_{i}^{r}=\left(u_{i}^{r},v_{i}^{r},w_{i}\right)\in \Delta_{i}$ let $V_{t_{i}^{r}}$ be the set of vertices that are excluded from those *W* with $u_{i}^{r},v_{i}^{r}\in W$ due to Observation 3.

In Lemma 6, we will show that any two triangles in Δ_1_ and Δ_2_ are vertex‐disjoint. Therefore, for each $t_{i}^{r}\in \Delta_{i}$, every vertex in $V_{t_{i}^{r}}$ is not contained in any triangle of $\Delta_{3-i}$. This implies that for any pair of triangles $t_{1}\in \Delta_{1},t_{2}\in \Delta_{2}$ the sets $V_{t_{1}},V_{t_{2}}$ are disjoint. Altogether, this means that we can bound the number of maximal transitive vertex sets $W⊇V^{\prime }$ not containing $w_{1},w_{2}$ from above by

(2) $$\begin{array}{ccc} & & \sum_{t=\left(w_{1},u_{1},u_{2}\right)\in \Delta_{1}}\sum_{t^{\prime }=\left(w_{2},u_{1}^{\prime },u_{2}^{\prime }\right)\in \Delta_{2}}M\left(T-\left\{w_{1},w_{2}\right\}-V_{t}-V_{t^{\prime }},V^{\prime }\cup \left\{u_{1},u_{2},u_{1}^{\prime },u_{2}^{\prime }\right\}\right)\\ \;\;\;\; & \leq & \sum_{t\in \Delta_{1}}\sum_{t^{\prime }\in \Delta_{2}}\beta^{n-2-|V_{t}\left|-\right|V_{t^{\prime }}|-\left(k+4\right)}\leq \beta^{n-k-6}\underset{\left(★\right)}{\underset{︸}{\sum_{t\in \Delta_{1}}\beta^{-|V_{t}|}\sum_{t^{\prime }\in \Delta_{2}}\beta^{-|V_{t^{\prime }}|}}}. \end{array}$$

Thus, our goal is now to bound (⋆). Fix i∈{1,2}. Let $t_{i}^{1},\cdots ,t_{i}^{|\Delta_{i}|}$ be an ordering of the triangles in $\Delta_{i}$ such that $|V_{t_{i}^{r}}\left|\leq \right|V_{t_{i}^{s}}|$ for $1\leq r<s\leq |\Delta_{i}|$. Then for any pair $r,s\in \{1,\cdots ,\Delta_{i}\}$ with r≠s, Observation 3 implies

$$|V_{t_{i}^{s}}\cap \left\{u_{i}^{r},v_{i}^{r}\right\}\left|+\right|V_{t_{i}^{r}}\cap \left\{u_{i}^{s},v_{i}^{s}\right\}|=2.$$

Thus, for any r<s, since β≥1, we get

$$\begin{array}{ccc} \beta^{-|V_{t_{i}^{s}}|}+\beta^{-|V_{t_{i}^{r}}|} & \leq & \beta^{-(|V_{t_{i}^{s}}\cup \left\{u_{i}^{r},v_{i}^{r}\right\}|)}+\beta^{-(|V_{t_{i}^{r}}\setminus \left\{u_{i}^{s},v_{i}^{s}\right\}|)}. \end{array}$$

Thus, we can bound (⋆) by the case where for any r<s,

$$\left|V_{t_{i}^{s}}\cap \left\{u_{i}^{r},v_{i}^{r}\right\}\right|=2\wedge \left|V_{t_{i}^{r}}\cap \left\{u_{i}^{s},v_{i}^{s}\right\}\right|=0.$$

Hence, we can assume that $|V_{t_{i}^{r}}|=2\left(r-1\right)$ for all $r=1,\cdots ,|\Delta_{i}|$. We obtain

$$\begin{array}{c} \sum_{t\in \Delta_{i}}\beta^{-|V_{t}|}\leq \sum_{r=0}^{|\Delta_{i}|-1}\beta^{-2r}\leq \sum_{r=0}^{\infty }\beta^{-2r}=\frac{\beta^{2}}{\beta^{2}-1}. \end{array}$$

Consequently, (⋆) is bounded by $\left(\frac{\beta^{2}}{\beta^{2}-1}\right)\cdot \left(\frac{\beta^{2}}{\beta^{2}-1}\right)=\frac{\beta^{4}}{{\left(\beta^{2}-1\right)}^{2}}$.

Let us now prove that indeed any triangle in Δ_1_ is disjoint from every triangle in Δ_2_.

Lemma 6 Let $v_{0},w_{1},w_{2},\Delta_{1}$, and Δ_2_ be defined as before. Then any triangle in Δ_1_ is vertex‐disjoint from every triangle in Δ_2_.

First note that $V^{\prime }$ is a transitive set, as Case 1 does not apply. Thus, the vertices in $V^{\prime }$ admit a topological order such that $v_{x}^{\prime }\to v_{y}^{\prime }$ for all $v_{x}^{\prime },v_{y}^{\prime }\in V^{\prime }$ with x>y. Second, for each vertex $z\in V\left(T\right)\setminus V^{\prime }$ the set $V^{\prime }\cup \left\{z\right\}$ is a transitive set, as Case 2 does not apply. Therefore, the vertices of $V\left(T\right)\setminus V^{\prime }$ can be partitioned into layers $Z_{1},\cdots ,Z_{\ell }$ such that for each $z\in Z_{r}$, $z\to v_{s}^{\prime }$ if and only if s<r.

We claim that for i=1,2, the vertices of any triangle $\left(u_{i}^{r},v_{i}^{r},w_{i}\right)\in \Delta_{i}$ all belong to the same layer. This implies in particular that for i=1,2, *all* vertices in triangles of $\Delta_{i}$ belong to the same layer. Since *w*_1_ and *w*_2_ are in different layers (as $v_{0}\to w_{1}$, $w_{2}\to v_{0}$), this shows that any triangle in Δ_1_ is vertex‐disjoint from any triangle in Δ_2_.

To show the claim, let i∈{1,2} and let $\left(u_{i}^{r},v_{i}^{r},w_{i}\right)\in \Delta_{i}$ be a triangle with $w_{i}\to u_{i}^{r},u_{i}^{r}\to v_{i}^{r},v_{i}^{r}\to w_{i}$. Suppose that $u_{i}^{r}\in Z_{u},v_{i}^{r}\in Z_{v},w_{i}\in Z_{w}$ for some u,v,w∈{1,⋯,l}. So we must show that u=v=w to prove the claim.

If u<w then $w_{i},u_{i}^{r},v_{u}^{\prime }$ form a triangle, contradicting that Case 4 does not apply. If v>w then $w_{i},v_{i}^{r},v_{v}^{\prime }$ form a triangle, again contradicting that Case 4 does not apply. Hence, v≤w≤u holds. If v<u then $u_{i}^{r},v_{i}^{r},v_{v}^{\prime }$ form a triangle, contradicting the definition of $\Delta_{i}$. So indeed u=v=w, and the claim holds. ▪

To complete the proof of Lemma 4, we must also consider those $W⊇V^{\prime }$ that contain exactly one of $w_{1},w_{2}$ (recall that at most one of $w_{1},w_{2}$ belongs to *W* as $v_{0}\in W$, so $w_{i}\in W$ implies $w_{3-i}\notin W$ for i=1,2). Overall, if Cases 1–5 do not apply, with the obtained bound on (⋆), by (2) we have

$$\begin{array}{ccc} M(T,V^{\prime }) & \leq & M\left(T-\left\{w_{1}\right\},V^{\prime }\cup \left\{w_{2}\right\}\right)+M\left(T-\left\{w_{2}\right\},V^{\prime }\cup \left\{w_{1}\right\}\right)+\beta^{n-6-k}\cdot \frac{\beta^{4}}{{\left(\beta^{2}-1\right)}^{2}}\\ & \leq & 2\cdot M\left(n-1,k+1\right)+\beta^{n-6-k}\cdot \frac{\beta^{4}}{{\left(\beta^{2}-1\right)}^{2}}\leq 2\cdot \beta^{n-k-2}+\frac{\beta^{n-k-2}}{{\left(\beta^{2}-1\right)}^{2}}. \end{array}$$

The last expression on the right‐hand side is at most $\beta^{n-k}$, since β≥1.5703.

This completes the proof of Lemma 4.

## 5 PROOF OF LEMMA 5

We will show by induction that $M\left(n\right)\leq 1.5949^{n}$ for all n∈N. For sake of contradiction, suppose that the statement of the lemma is not true. Let $T\in T_{n}$ be a counterexample with minimum number of vertices. By Observation 1, we can assume that *T* is strong. In particular, any vertex of *T* has out‐degree at most n−2.

First suppose that *T* satisfies the following stronger restriction:

(✠) AnyvertexofThasout−degreeatmostn−6.

Then we know by Lemma 3, that either *T* is regular, or for any d∈N at most 2*d* vertices have out‐degree at least n−d−1.

If *T* is regular, then any vertex has out‐degree exactly (n−1)/2. Using that $M\left(T\right)\leq \sum_{v\in V(T)}M\left(d^{+}\left(v\right)\right)$ by Lemma 2, it follows that

$$M\left(T\right)\leq \sum_{v\in V(T)}M\left(d^{+}\left(v\right)\right)\leq n\cdot M\left(\left(n-1\right)/2\right)\leq n\cdot 1.5949^{\frac{n-1}{2}}.$$

For n≥11 we obtain $21^{n/7}>n\;\cdot 1.5949^{\frac{n-1}{2}}$, and so any regular tournament with at least 11 vertices has at most $21^{n/7}$ maximal transitive vertex sets. For n≤9, the inequality $M\left(n\right)\leq 21^{n/7}$ was shown explicitly by Gaspers and Mnich 6. This completes the proof of Theorem 2.

So we may assume that for any d∈N, at most 2*d* vertices have out‐degree at least n−d−1. Let $v_{1},\cdots ,v_{n}$ be a labeling of the vertices of *T* such that $d^{+}\left(v_{1}\right)\geq d^{+}\left(v_{2}\right)\geq \cdots \geq d^{+}\left(v_{n}\right)$. Then Lemma 3 implies that for d∈N,

$$d^{+}\left(v_{i}\right)\leq n-d-2\;\text{for}\;i>2d.$$

Choosing $d=\left\lfloor \frac{i-1}{2}\right\rfloor$ implies i>2d, and we obtain

(3) $$d^{+}\left(v_{i}\right)\leq n-\left\lfloor \frac{i-1}{2}\right\rfloor -2\;\text{for}\;i=1,\cdots ,n.$$

It follows that

$$\begin{array}{ccc} M(T) & \leq & \sum_{i=1}^{n}M\left(d^{+}\left(v_{i}\right)\right)\;\;\text{(Lemma}\;\text{2)}\\ & \leq & \sum_{i=1}^{10}M\left(d^{+}\left(v_{i}\right)\right)+\sum_{i=11}^{n}M\left(n-\left\lfloor \frac{i-1}{2}\right\rfloor -2\right)\;\;\text{(by}\;\text{(3))}\\ & \leq & 10\cdot M\left(n-6\right)+\sum_{i=11}^{n}\beta^{n-\left\lfloor \frac{i-1}{2}\right\rfloor -2}\\ & \leq & 10\cdot \beta^{n-6}+\beta^{n-6}\sum_{i=1}^{\infty }\beta^{-\left\lfloor \frac{i-1}{2}\right\rfloor -1}\\ & \leq & 10\cdot \beta^{n-6}+\beta^{n-6}\cdot 2\cdot \sum_{i=1}^{\infty }\beta^{-i}\\ & = & 10\cdot \beta^{n-6}+2\cdot \frac{\beta^{n-6}}{\beta -1}, \end{array}$$

where the last expression is bounded by $\beta^{n}$ since β≥1.5462. This completes the analysis of tournaments *T* satisfying (✠).

We will now work toward removing assumption (✠). This amounts to bounding M(T) in each of the four cases $\Delta^{+}\left(T\right)=n-i$ for i=2,3,4,5. In each case, we bound M(T) via $M(\tilde{n})$ for values $\tilde{n}<n$ and the result of Lemma 4. With each case (and subcase thereof) we associate a *branching vector* $b=(b_{1},\cdots ,b_{t})$, where *t* is the number of branches we consider of how a particular maximal transitive vertex set *W* of *T* can look like. Each number $b_{i},i=1,\cdots ,t$ is a positive integer that is the sum of Δn and Δk, where Δn is the number of vertices by which the tournament order *decreases* in that branch and Δk is the number of vertices by which the parameter *k* *increases*. It might be that one of Δn and Δk is equal to zero in some branch, but the sum $b_{i}=\Delta n+\Delta k$ is always positive. We will show that $M\left(T\right)\leq \sum_{i=1}^{t}\beta^{n-b_{i}}\leq \beta^{n}$ in each case.

To bound of M(T), we classify all maximal transitive vertex sets of *T*. Let *W* be a maximal transitive vertex set of *T*. We branch on carefully selected vertices whether they belong to *W* or not. These choices either exclude certain other vertices from *W* (by acyclicity of *W*) and thus yield Δn>0; they force certain other vertices to be included into *W*, based on the following observation:

Observation 4 Let *W* be a maximal transitive vertex set of *T*, and let *v* be a vertex of *T*. If v∉W then at least one predecessor of *v* in *T* belongs to *W*. Equivalently, if no predecessor of *v* belongs to *W*, then v∈W.

We will apply this observation with various choices for *v*. These choices and their implications will be depicted by case trees that also show the pair (Δn,Δk) for each branch.

**Case 1**: $\Delta^{+}\left(T\right)=n-2$
  Let $v^{★}$ be a vertex with maximum out‐degree and unique predecessor *b*. We distinguish the following subcases.
  **Case 1.1**: $d^{+}\left(b\right)=n-2$
    Let $\tilde{b}$ be the only predecessor of *b*. Let *W* be a maximal transitive vertex set of *T*.
    When b∉W, we have that $v^{★}\in W$ by applying Observation 4 with $v=v^{★}$. We further have that $\tilde{b}\in W$ by applying Observation 4 with v=b.
    This shows that *W* can be categorized as follows, with branching pairs (Δn,Δk):

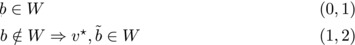

    This yield a branching vector b=(1,3), which solves to 1.4656.
  **Case 1.2**: $d^{+}\left(b\right)\leq n-3$
    Let *W* be a maximal transitive vertex set of *T*. As in Case 1.1, at least one of $v^{★},b$ belongs to *W*, by Observation 4. If $v^{★},b\in W$ then no predecessor of *b* belongs to *W*, as T[W] is acyclic. Since *b* has at least two predecessors, these observations yield:

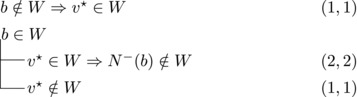

  This yields a branching vector of (2, 4, 2), which solves to 1.5538.
  This completes the analysis of Case 1, where we used that β≥1.5538.
**Case 2**: $\Delta^{+}\left(T\right)=n-3$
  Let $V^{★}$ be the set of vertices with maximum out‐degree.

Lemma 7 Either some vertex $v^{★}\in V^{★}$ has two predecessors of out‐degree exactly n−3 or two predecessors of out‐degree at most n−4, or $V^{★}$ induces a triangle.

By Lemma 3, $V^{★}$ contains at most four vertices.

If $V^{★}$ contains a single vertex $v^{★}$ then its two predecessors are outside $V^{★}$, and thus have out‐degree at most n−4.

If $V^{★}$ consists of two vertices $v_{1}^{★},v_{2}^{★}$ then assume $v_{1}^{★}\to v_{2}^{★}$. Thus, the two predecessors of $v_{1}^{★}$ are outside $V^{★}$, and thus have out‐degree at most n−4.

If $V^{★}$ consists of three vertices $v_{1}^{★},v_{2}^{★},v_{3}^{★}$ then we may assume that $V^{★}$ does not induce a triangle (else, we are done). Thus, we can assume that $v_{i}^{★}\to v_{j}^{★}$ for 1≤i<j≤3. Thus, the two predecessors of $v_{1}^{★}$ are outside $V^{★}$, and thus have out‐degree at most n−4.

Finally, assume that $V^{★}$ consists of four vertices. Then the subtournament $T[V^{★}]$ has exactly six arcs. As each vertex in $V^{★}$ has two incoming arcs, this implies that for some vertex in $V^{★}$ its two predecessors are also in $V^{★}$ and therefore have out‐degree n−3. ▪

Therefore, we can distinguish whether

- some $v^{★}\in V^{★}$ has two predecessors with out‐degree exactly n−3 (Case 2.1),
- or some $v^{★}\in V^{★}$ has two predecessors with out‐degree at most n−4 (Cases 2.2–2.7),
- or *T* has exactly three vertices with out‐degree n−3 that form a triangle (Case 2.8).

In Cases 2.1–2.7, let $N^{-}\left(v^{★}\right)=\left\{b_{1},b_{2}\right\}$ and $b_{1}\to b_{2}$.

**Case 2.1**: $d^{+}\left(b_{1}\right)=n-3$ and $d^{+}\left(b_{2}\right)=n-3$
  Then *b*_1_ and *b*_2_ have at most one common predecessor. Let $N^{-}\left(b_{1}\right)=\left\{u_{1},u_{2}\right\}$.
  **Case 2.1.1**: $N^{-}\left(b_{1}\right)\cap N^{-}\left(b_{2}\right)=\emptyset$.
  Let $N^{-}\left(b_{2}\right)=\left\{b_{1},u_{3}\right\}$ for some $u_{3}\notin N^{-}\left(b_{1}\right)$. Note that if $v^{★}\notin W,b_{2}\in W$, then $u_{3}\in W$. (If $b_{1}\in W$, $u_{1},u_{2}\notin W$ since $b_{1},b_{2}$, and $u_{i}$ are forming a triangle for i=1,2. Due to the maximality of *W*, the addition of $v^{★}$ has to generate a triangle and therefore $u_{3}\in W$.) Then we branch as follows:

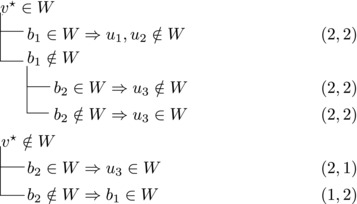

  This yields a branching vector of (4, 4, 4, 3, 3) that solves to 1.5748.
  Case 2.1.2 $N^{-}\left(b_{1}\right)\cap N^{-}\left(b_{2}\right)=\left\{u_{2}\right\}$.

Then we branch as follows:


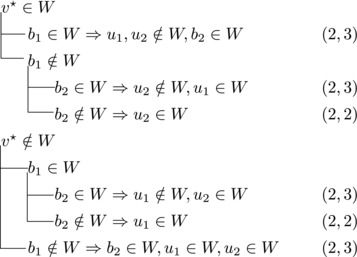


This yields a branching vector of (5, 5, 4, 5, 4, 5) that solves to 1.4736.

**Case 2.2**: $d^{+}\left(b_{1}\right)\leq n-4$, $d^{+}\left(b_{2}\right)\leq n-4$ and $b_{1},b_{2}$ share no common predecessor.
  Any predecessor of *b*_1_ forms a triangle with $b_{1},b_{2}$ and therefore any maximal transitive vertex set containing both $b_{1},b_{2}$ cannot contain any predecessor of *b*_1_. Then we branch as follows:

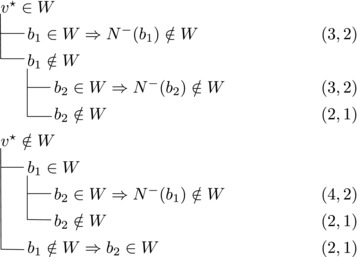

  This yields a branching vector of (5, 5, 3, 6, 3, 3) that solves to 1.5923.
**Case 2.3**: $d^{+}\left(b_{1}\right)\leq n-4$, $d^{+}\left(b_{2}\right)=n-4$ and one predecessor of *b*_2_ is a predecessor of *b*_1_
  Let $N^{-}\left(b_{1}\right)=\left\{w_{1},...,w_{l}\right\}$ with l≥3 and let $N^{-}\left(b_{2}\right)=\left\{b_{1},u,w_{1}\right\}$, so that *w*_1_ is the unique predecessor common to $b_{1},b_{2}$.
  **Case 2.3.1**: $w_{i}\to u$ for some $i\in \left\{2,...,l\right\}$.
  Then $\left\{u,b_{2},w_{i}\right\}$ induces a triangle.
  Then we branch as follows:

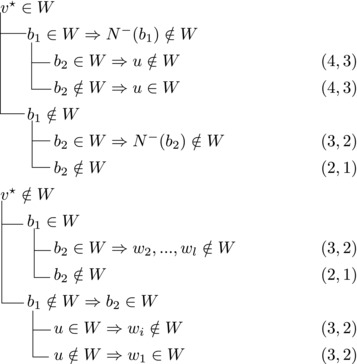

  This yields a branching vector of (7, 7, 5, 3, 5, 3, 5, 5) that solves to 1.5780.
  **Case 2.3.2**: $u\to w_{i}$ for all $i\in \left\{2,\ldots ,l\right\}$
    Then $\left\{u,w_{i},b_{1}\right\}$ induces a triangle for all $i\in \left\{2,\ldots ,l\right\}$.
    Then we branch as follows:

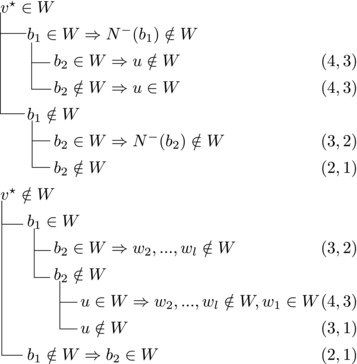

  This yields a branching vector of (7, 7, 5, 3, 5, 7, 4, 3) that solves to 1.5772.
**Case 2.4**: $d^{+}\left(b_{1}\right)=n-4$, $d^{+}\left(b_{2}\right)=n-4$ and all predecessors of *b*_2_ other than *b*_1_ are predecessors of *b*_1_
  Let *u* be the only predecessor of *b*_1_ that is not a predecessor of *b*_2_. Then we branch as follows:

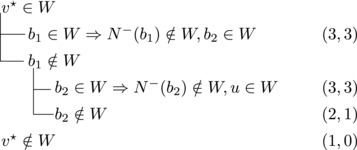

  This yields a branching vector of (6, 6, 3, 1) that solves to 1.5912.
**Case 2.5**: $d^{+}\left(b_{1}\right)\leq n-5,d^{+}\left(b_{2}\right)=n-4$ and all predecessors of *b*_2_ are predecessors of *b*_1_
  Then we branch as follows:

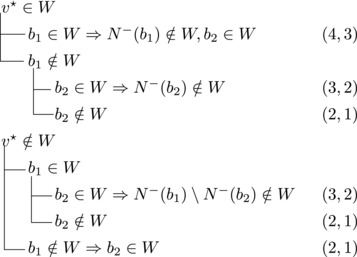

  This yields a branching vector of (7, 5, 3, 5, 3, 3) that solves to 1.5820.
**Case 2.6**: $d^{+}\left(b_{1}\right)=n-4$ and $d^{+}\left(b_{2}\right)\leq n-5$
  We already considered that $b_{1},b_{2}$ have no common predecessor in Case 2.2. Hence, $b_{1},b_{2}$ have either one, two, or three common predecessors.
  **Case 2.6.1**: *b*_1_ and *b*_2_ have exactly one common predecessor.
    Then we branch as follows:

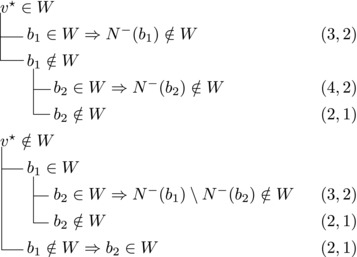

  This yields a branching vector of (5, 6, 3, 5, 3, 3) that solves to 1.5923.
  **Case 2.6.2**: *b*_1_ and *b*_2_ share exactly two predecessors.
    Then either $s_{b_{2}}<n-5$, or *b*_2_ has exactly one predecessor that is not a predecessor of *b*_1_. Let *u*_1_ be the only predecessor of *b*_1_ that is not a predecessor of *b*_2_. Note that any maximal transitive vertex set that contains $n,b_{2}$ but not *b*_1_ has to contain *u*_1_.
  For $s_{b_{2}}<n-5$ we branch as follows:

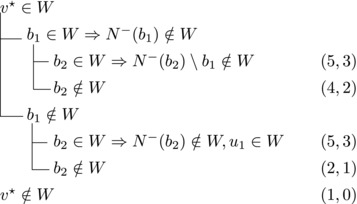

  This yields a branching vector of (8, 6, 8, 3, 1) that solves to 1.5817.
  For $s_{b_{2}}=n-5$ and *u*_2_ being the only predecessor of *b*_2_ that is not a predecessor of *b*_1_, we branch as follows:

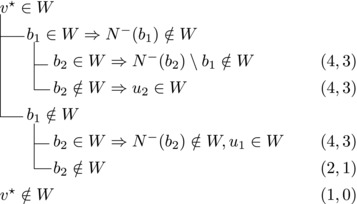

  This yields a branching vector of (7, 7, 7, 3, 1) that solves to 1.5861.
  **Case 2.6.3**: All predecessors of *b*_1_ are predecessors of *b*_2_.
    Then any maximal transitive vertex set containing $v^{★},b_{2}$ also contains *b*_1_. Then we branch as follows:

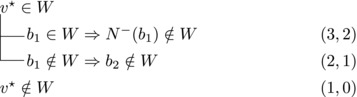

  This yields a branching vector of (5, 3, 1) that solves to 1.5702.
**Case 2.7**: $d^{+}\left(b_{1}\right)\leq n-5$ and $d^{+}\left(b_{2}\right)\leq n-5$
  Then we branch as follows:

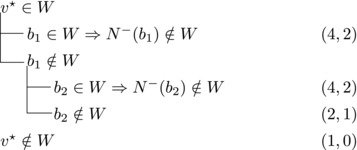

  This yields a branching vector of (6, 6, 3, 1) that solves to 1.5912.
**Case 2.8**: There are exactly three vertices with score n−3 and they form a triangle.

Call these vertices $v_{1},v_{2},v_{3}$ and assume $v_{1}\to v_{2}$, $v_{2}\to v_{3}$, and $v_{3}\to v_{1}$.

Suppose that $v_{1},v_{2},v_{3}$ have distinct predecessors, and let $b_{i}$ be the predecessor of $v_{i}$.

Note that $v_{i},v_{i+1},b_{i}$ form a triangle and that $b_{i+1}$ is the only predecessor of $v_{i+1}$ other than $v_{i}$. Therefore, we branch as follows:


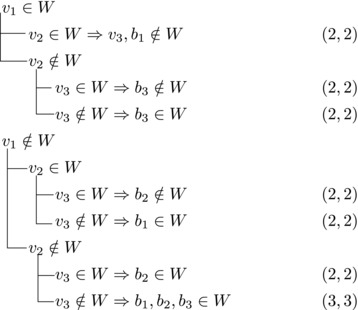


This yields a branching vector of (4, 4, 4, 4, 4, 4, 6) that solves to 1.5903.

Now suppose that $v_{1},v_{2}$ have a common predecessor *b* and *b*_3_ is the predecessor of *b*_3_.

Since *v*_3_ is the only vertex where the orientations of the arcs incident to *v*_1_ differ from those incident to *v*_2_, any maximal transitive vertex set that does not contain *v*_3_ either contains both $v_{1},v_{2}$ or none of them. We branch as follows:


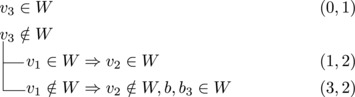


This yields a branching vector of (1, 3, 5) that solves to 1.5702.

If all three vertices have a common predecessor *b*, any maximal transitive vertex set that contains some vertex of $v_{1},v_{2},v_{3}$ contains at exactly two of them. We branch as follows:


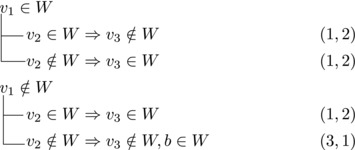


This yields a branching vector of (3, 3, 3, 4) that solves to 1.5397.

**Case 3**: $\Delta^{+}\left(T\right)=n-4$
  Let $V^{★}$ be the set of vertices with out‐degree n−4. Since *T* is strong, some vertex $v^{★}\in V^{★}$ has a predecessor in $V\left(T\right)\setminus V^{★}$. Let $b_{1},b_{2},b_{3}$ be the predecessors of $v^{★}$. For i=1,2,3 let ${\tilde{s}}_{i}=\left|N^{-}\left(b_{i}\right)\setminus \left\{b_{1},b_{2},b_{3}\right\}\right|$; assume, without loss of generality, that ${\tilde{s}}_{1}\geq {\tilde{s}}_{2}\geq {\tilde{s}}_{3}$.
  By the choice of $v^{★}$ we have $|N^{-}\left(b_{1}\right)\left|+\right|N^{-}\left(b_{2}\right)\left|+\right|N^{-}\left(b_{3}\right)|\geq 10$ and hence ${\tilde{s}}_{1}+{\tilde{s}}_{2}+{\tilde{s}}_{3}\geq 7$. Note that $|N^{-}\left(b_{i}\right)|\geq 3$ for i=1,2,3 and $|N^{-}\left(b_{j}\right)|\geq 4$ for some j∈{1,2,3}. Therefore ${\tilde{s}}_{1}\geq 3$, ${\tilde{s}}_{2}\geq 2$, ${\tilde{s}}_{3}\geq 1$.
  We will estimate bounds for different values of ${\tilde{s}}_{1},{\tilde{s}}_{2},{\tilde{s}}_{3}$.
  **Case 3.1**: ${\tilde{s}}_{3}=1$
  If ${\tilde{s}}_{3}=1$, then either ${\tilde{s}}_{2}\geq 3$ or ${\tilde{s}}_{1}\geq 4$. Let $\left\{u\right\}=N^{-}\left(b_{3}\right)\setminus \left\{b_{1},b_{2},b_{3}\right\}$. Then we branch as follows:

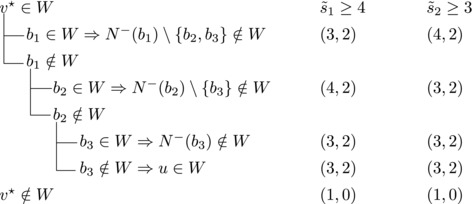

  This yields a branching vector of (5, 6, 5, 5, 1) that solves to 1.5812.
  **Case 3.2**: ${\tilde{s}}_{1}\geq 4$ or ${\tilde{s}}_{2}\geq 3$, ${\tilde{s}}_{3}\geq 2$
    We branch as follows:

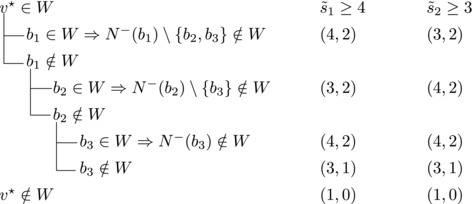

  This yields a branching vector of (6, 5, 6, 4, 1) that solves to 1.5949.
  Since ${\tilde{s}}_{2}\geq {\tilde{s}}_{3}$, we can now assume that ${\tilde{s}}_{1}=3,{\tilde{s}}_{2}=2,{\tilde{s}}_{3}=2$.
  **Case 3.3**: ${\tilde{s}}_{1}=3,{\tilde{s}}_{2}=2,{\tilde{s}}_{3}=2$ and all predecessors of *b*_2_ except the $b_{i}$ are predecessors of *b*_3_
    We branch as follows:

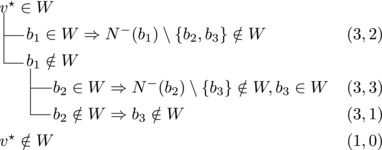

  This yields a branching vector of (5, 6, 4, 1) that solves to 1.5516.
  **Case 3.4**: ${\tilde{s}}_{1}=3,{\tilde{s}}_{2}=2,{\tilde{s}}_{3}=2$, and $b_{2},b_{3}$ have exactly one common predecessor *u*_1_ in $V\left(T\right)\setminus \left\{b_{1},b_{2},b_{3}\right\}$
    Let *W* be a maximal transitive vertex set, $u_{2}\neq u_{1}$ be the other predecessor of *b*_2_ and $u_{3}\neq u_{1}$ be the one of *b*_3_. If $v^{★}\in W$ but $b_{2},b_{3}\notin W$, then either $u_{1}\in W$ or $u_{2},u_{3}\in W$, as otherwise *b*_2_ or *b*_3_ could be added to *W*. Therefore, we branch as follows:

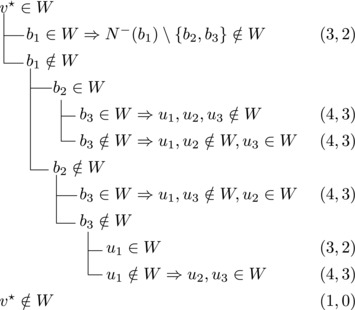

  This yields a branching vector of (5, 7, 7, 7, 5, 7, 1) that solves to 1.5793.
  Henceforth, we can assume that $b_{2},b_{3}$ have no common predecessor except possibly *b*_1_.
  **Case 3.5**: ${\tilde{s}}_{1}=3,{\tilde{s}}_{2}=2,{\tilde{s}}_{3}=2$, $b_{2},b_{3}$ have no common predecessor in $V\left(T\right)\setminus \left\{b_{1},b_{2},b_{3}\right\}$ and two predecessors of *b*_1_ in $V\left(T\right)\setminus \left\{b_{1},b_{2},b_{3}\right\}$ are predecessors of $b_{i}$ for some i=2,3
    Assume, without loss of generality, that $b_{3}=b_{i}$ and let *u*_1_ be the only predecessor of *b*_1_ that is not a predecessor of *b*_3_. Since ${\tilde{s}}_{1}=3$, there is at least one predecessor of *b*_2_ that is not a predecessor of *b*_1_. We branch as follows:

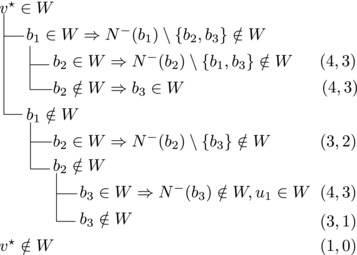

  This yields a branching vector of (7, 7, 5, 7, 4, 1) that solves to 1.5904.
  **Case 3.6**: ${\tilde{s}}_{1}=3,{\tilde{s}}_{2}=2,{\tilde{s}}_{3}=2$, $b_{2},b_{3}$ have no common predecessor in $V\left(T\right)\setminus \left\{b_{1}\right\}$ and both *b*_2_ and *b*_3_ share at most one predecessor of *b*_1_.
    There are three cases to consider:
    - *b*_1_ shares no predecessor in $V\left(T\right)\setminus \left\{b_{1},b_{2},b_{3}\right\}$ with *b*_2_ and none with *b*_3_, or
    - *b*_1_ shares one predecessor with each of *b*_2_ and *b*_3_, or
    - *b*_1_ shares a predecessor with one of $b_{2},b_{3}$ but none with the other; without loss of generality, let *b*_1_ share a predecessor with *b*_3_.
    We will split each of the cases into two subcases where we consider if $b_{1},b_{2}$, and *b*_3_ are forming a triangle.
    **Case 3.6.1**: *b*_1_ shares one predecessor in $V\left(T\right)\setminus \left\{b_{1},b_{2},b_{3}\right\}$ with *b*_2_ and one with *b*_3_.
$b_{1},b_{2}$, and *b*_3_ do not form a triangle.
      Let *w*_1_ the common predecessor of *b*_1_ and *b*_2_ and let *w*_2_ be the common predecessor of *b*_1_ and *b*_3_. For i=1,2,3 let $u_{i}$ be the predecessor of $b_{i}$ that is neither *w*_1_ nor *w*_2_. We branch as follows:

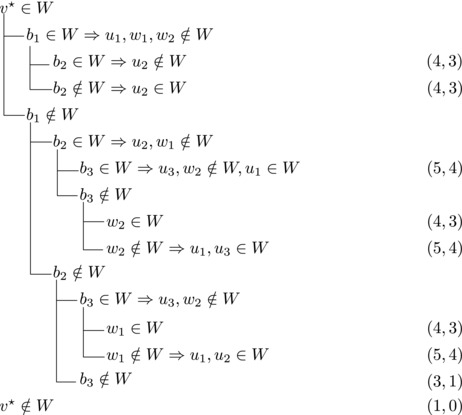

      This yields a branching vector of (7, 7, 9, 7, 9, 7, 9, 4, 1) that solves to 1.5828.
    **Case 3.6.2**: *b*_1_ shares one predecessor in $V\left(T\right)\setminus \left\{b_{1},b_{2},b_{3}\right\}$ with *b*_2_ and one with *b*_3_.
$b_{1},b_{2}$, and *b*_3_ form a triangle.
      Let $u_{1},u_{2},u_{3},w_{1},w_{2}$ be as in Case 3.6.1. Then we branch as follows:

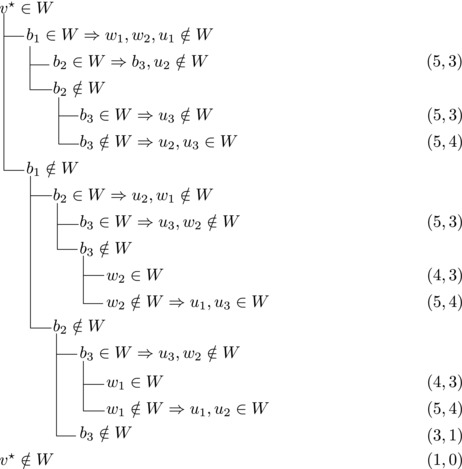

      This yields a branching vector of (8, 8, 9, 8, 7, 9, 7, 9, 4, 1) that solves to 1.5805.
    **Case 3.6.3**: *b*_1_ shares no predecessor in $V\left(T\right)\setminus \left\{b_{1},b_{2},b_{3}\right\}$ with *b*_2_ and one with *b*_3_.
$b_{1},b_{2}$, and *b*_3_ do not form a triangle.
      Let *u*_3_ the only predecessor of *b*_3_ that is not a predecessor of *b*_1_. Then we branch as follows:

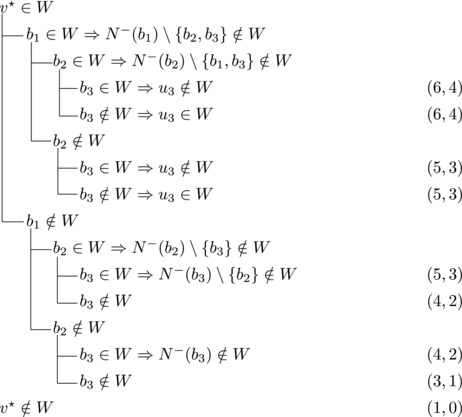

      This yields a branching vector of (10, 10, 8, 8, 8, 6, 6, 4, 1) that solves to 1.5912.
    **Case 3.6.4**: *b*_1_ shares no predecessor in $V\left(T\right)\setminus \left\{b_{1},b_{2},b_{3}\right\}$ with *b*_2_ and one with *b*_3_.
$b_{1},b_{2}$, and *b*_3_ form a triangle.
      Let *u*_3_ be as defined in Case 3.6.3. Then we branch as follows:
       
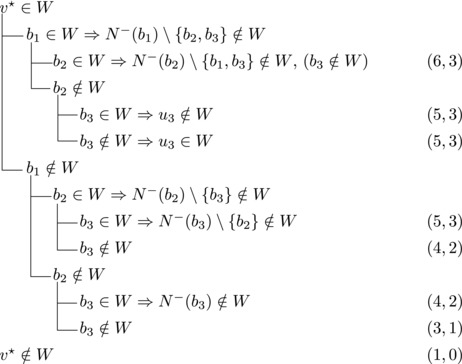

      This yields a branching vector of (9, 8, 8, 8, 6, 6, 4, 1) that solves to 1.5889.
    **Case 3.6.5**: *b*_1_ shares no predecessor in $V\left(T\right)\setminus \left\{b_{1},b_{2},b_{3}\right\}$ with *b*_2_ and none with *b*_3_.
$b_{1},b_{2}$, and *b*_3_ do not form a triangle.
      Since ${\tilde{s}}_{2}={\tilde{s}}_{3}=2$ but $|N^{-}\left(b_{i}\right)|\leq n-4$ for i=2,3, there is a vertex $b_{j}\neq b_{i}$, j=1,2,3 such that $b_{j}\to b_{i}$. As the vertices $b_{1},b_{2}$, and *b*_3_ form a transitive set, we have $b_{1}\to b_{2}$ and $b_{1}\to b_{3}$. Let $u_{1},u_{2},u_{3}$ the predecessors of *b*_1_. Note that $b_{2},b_{3},v^{★}$ and each $u_{i}$ for i=1,2,3 form a cycle with *b*_1_. Therefore, if *W* contains some $u_{i}$, it can not contain $b_{2},b_{3}$, or $v^{★}$. If it contains $b_{2},b_{3}$, or $v^{★}$, it can not contain any $u_{i}$. Thus, we branch as follows:
       
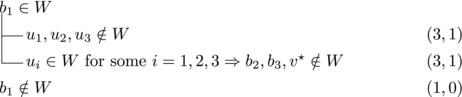

      This yields a branching vector of (4, 4, 1) that solves to 1.5437.
    **Case 3.6.6**: *b*_1_ shares no predecessor in $V\left(T\right)\setminus \left\{b_{1},b_{2},b_{3}\right\}$ with *b*_2_ and none with *b*_3_.

$b_{1},b_{2}$, and *b*_3_ form a triangle. Assume without loss of generality that $b_{1}\to b_{2},b_{2}\to b_{3}$ and $b_{3}\to b_{1}$. Therefore all predecessors of *b*_1_ form a triangle with *b*_1_ and *b*_2_ and all predecessors of *b*_3_ form a triangle with *b*_3_ and *b*_1_. We branch as follows:

                          
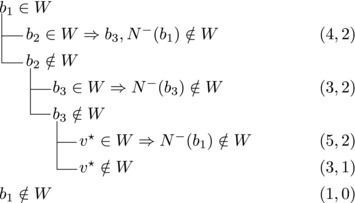


This yields a branching vector of (6, 5, 7, 4, 1) that solves to 1.5800.

This completes the analysis of Case 3.

**Case 4**: $\Delta^{+}\left(T\right)=n-5$

Let $V^{★}$ be the set of vertices with maximum out‐degree; Since *T* is strong, there is a vertex $v^{★}$ that has a predecessor in $V\left(T\right)\setminus V^{★}$.

Let $N^{-}\left(v^{★}\right)=\left\{b_{1},b_{2},b_{3},b_{4}\right\}$. By the choice of $v^{★}$, the vertices $b_{1},b_{2},b_{3},b_{4}$ together have at least 17 incoming arcs. Exactly 6 of these arcs are incident to two of $b_{1},b_{2},b_{3},b_{4}$. The remaining 11 arcs are incoming from vertices in $V\left(T\right)\setminus \left\{b_{1},b_{2},b_{3},b_{4}\right\}$. For i=1,⋯,4 let ${\tilde{s}}_{i}$ be the number of arcs incoming to $b_{i}$ from $V\left(T\right)\setminus \left\{b_{1},b_{2},b_{3},b_{4}\right\}$, i.e. ${\tilde{s}}_{i}=\left|N^{-}\left(b_{i}\right)\setminus \left\{b_{1},b_{2},b_{3},b_{4}\right\}\right|$. Assume, without loss of generality, that ${\tilde{s}}_{1}\geq {\tilde{s}}_{2}\geq {\tilde{s}}_{3}\geq {\tilde{s}}_{4}$.

Observation 5 It holds, ${\tilde{s}}_{4}\geq 1$ and ${\tilde{s}}_{3}\geq 2$. If ${\tilde{s}}_{2}=2$, then ${\tilde{s}}_{3}={\tilde{s}}_{4}=2$.

Since $|N^{-}\left(b_{i}\right)|\geq 4$ for all i=1,2,3,4, we get $|N^{-}\left(b_{i}\right)\setminus \left\{b_{1},b_{2},b_{3},b_{4}\right\}|\geq 1$. There is at most one vertex with with three incoming arcs from $b_{1},b_{2},b_{3},b_{4}$. This yields ${\tilde{s}}_{3}\geq 2$.

Assume that ${\tilde{s}}_{2}={\tilde{s}}_{3}=2$ and ${\tilde{s}}_{4}=1$. If ${\tilde{s}}_{4}=1$, then $b_{1}\to b_{4}$, $b_{2}\to b_{4}$, and $b_{3}\to b_{4}$. Since ${\tilde{s}}_{3}=2$, $b_{1}\to b_{3}$, and $b_{2}\to b_{3}$. But then we get ${\tilde{s}}_{2}\geq 3$ that is a contradiction. ▪

This leaves exactly six feasible vectors $\left({\tilde{s}}_{1},{\tilde{s}}_{2},{\tilde{s}}_{3},{\tilde{s}}_{4}\right)$; see Table 2. We give estimates for M(T) in terms of pairs (Δn,Δk) for each of these six vectors in Table 2.

**Table 2:** The 6 possibilities for (${\tilde{s}}_{1},{\tilde{s}}_{2},{\tilde{s}}_{3},{\tilde{s}}_{4}$)

$\left({\tilde{s}}_{1},{\tilde{s}}_{2},{\tilde{s}}_{3},{\tilde{s}}_{4}\right)$	(5,3,2,1)	(5,2,2,2)	(4,4,2,1)	(4,3,3,1)	(4,3,2,2)	(3,3,3,2)
$M(T-v^{★},\emptyset )$	(1, 0)	(1, 0)	(1, 0)	(1, 0)	(1, 0)	(1, 0)
$M(T,\left\{v^{★},b_{1}\right\})$	(5, 2)	(5, 2)	(4, 2)	(4, 2)	(4, 2)	(3, 2)
$M(T-\left\{b_{1}\right\},\left\{v^{★},b_{2}\right\})$	(4, 2)	(3, 2)	(5, 2)	(4, 2)	(4, 2)	(4, 2)
$M(T-\left\{b_{1},b_{2}\right\},\left\{v^{★},b_{3}\right\})$	(4, 2)	(4, 2)	(4, 2)	(5, 2)	(4, 2)	(5, 2)
$M(T-\left\{b_{1},b_{2},b_{3}\right\},\left\{v^{★},b_{4}\right\})$	(4, 2)	(5, 2)	(4, 2)	(4, 2)	(5, 2)	(5, 2)
$M(T-\left\{b_{1},b_{2},b_{3},b_{4}\right\},\left\{v^{★}\right\})$	(4, 1)	(4, 1)	(4, 1)	(4, 1)	(4, 1)	(4, 1)

To this end, note that any maximal transitive vertex set *W* that contains $v^{★},b_{i}$ cannot contain any predecessor of $b_{i}$ in $V\left(T\right)\setminus \left\{b_{1},b_{2},b_{3},b_{4},v^{★}\right\}$.

This yields for four of the cases

$$\begin{array}{ccc} M(T) & \leq & M(n-1)+M(n-4,1)+3\cdot M(n-4,2)+M(n-5,2)\\ & \leq & \beta^{n-1}+\beta^{n-5}+3\cdot \beta^{n-6}+\beta^{n-7}, \end{array}$$

where the last expression is bounded by $\beta^{n}$ since β≥1.5612.

For the remaining two cases, M(T) is bounded by

$$\begin{array}{ccc} M(T) & \leq & M(n-1)+M(n-4,1)+M(n-3,2)+M(n-4,2)+2\cdot M(n-5,2)\\ & \leq & \beta^{n-1}+2\cdot \beta^{n-5}+\beta^{n-6}+2\cdot \beta^{n-7}, \end{array}$$

where the last expression is bounded by $\beta^{n}$ since β≥1.5691.

This last case completes the proof of Lemma 5.

## 6 DISCUSSION

In this article, we narrowed the gap between the lower and upper bounds for the maximum number M(n) of minimal FVS in *n*‐vertex tournaments, to $1.5448^{n}\leq M\left(n\right)\leq 1.5949^{n}$. It remains to determine the growth of M(n) exactly—Gaspers and Mnich 6 conjectured that $M\left(n\right)\leq 21^{n/7}\approx 1.5448^{n}$ for all n∈N, and we repose this conjecture here.

In a different direction, it would be interesting to prove nontrivial upper bounds of the form $c^{n}$ for some constant c<2, on the number of minimal FVS in general directed graphs. As far as we know, currently only a bound of $2^{n}/\sqrt{n}$ is known, implied by Sperner's Lemma.
